# Neck fracture of a cementless forged titanium alloy femoral stem following total hip arthroplasty: a case report and review of the literature

**DOI:** 10.1186/1752-1947-1-174

**Published:** 2007-12-06

**Authors:** Theodoros B Grivas, Olga D Savvidou, Spyridon A Psarakis, Pierre-Francois Bernard, George Triantafyllopoulos, Ioannis Kovanis, Panagiotis Alexandropoulos

**Affiliations:** 1 Orthopaedic Department, "Thriasio" General Hospital, G. Gennimata Avenue, Magula, 19600, Greece

## Abstract

**Introduction:**

Fractures of the neck of the femoral component have been reported in uncemented total hip replacements, however, to our knowledge, no fractures of the neck of a cementless forged titanium alloy femoral stem coated in the proximal third with hydroxy-apatite have been reported in the medical literature.

**Case presentation:**

This case report describes a fracture of the neck of a cementless forged titanium alloy stem coated in the proximal third with hydroxy-apatite.

**Conclusion:**

The neck of the femoral stem failed from fatigue probably because of a combination of factors described analytically below.

## Introduction

Fracture of the femoral stem was a frequent complication of first-generation, forged stainless steel or cast cobalt chrome femoral components used for total hip arthroplasty. Charnley estimated a prevalence of 0.23%, although the prevalence was as high as 11% with other stem designs. With the development of femoral stems made of forged cobalt chromium-molybdenum or titanium alloys in the 1980s, this complication became rarer. Fractures of the neck of the femoral component have been reported in uncemented total hip replacements, however, to our knowledge, no fractures of the neck of a cementless forged titanium alloy femoral stem coated in the proximal third with hydroxy-apatite have been reported in the medical literature.

## Case presentation

**Figure 1:**
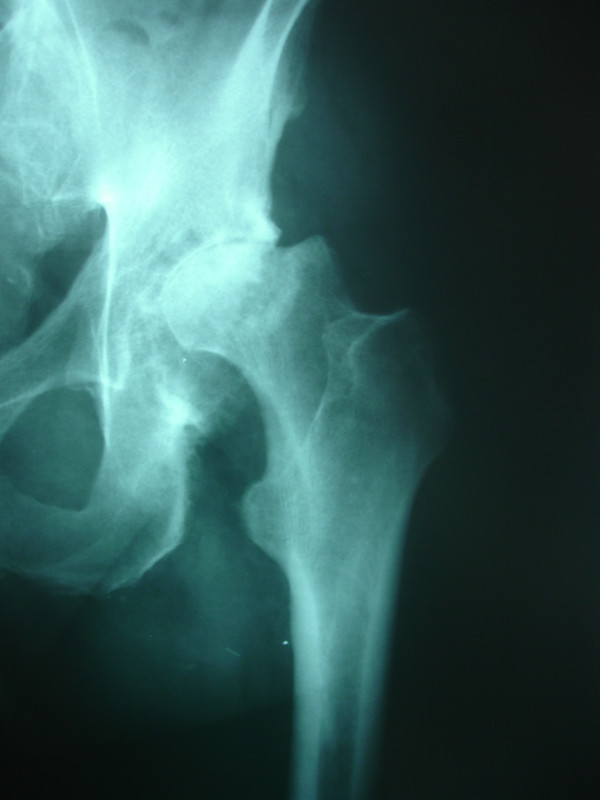
Preoperative anteroposterior radiograph of the left hip. Avascular necrosis of the left hip.

**Figure 2:**
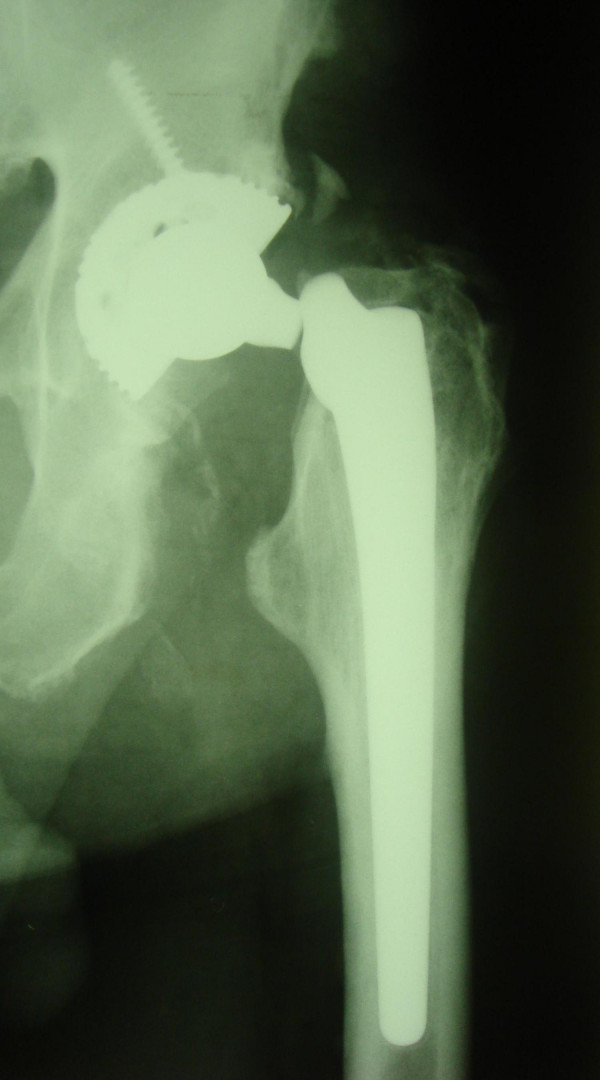
Fractured neck of the femoral implant prior to retrieval.

A 64 year-old man of weight 70 kg and height 1.65 m underwent a total hip arthroplasty (THA) in January 2003, due to severe osteoarthritis of his left hip following avascular osteonecrosis (figure 1). The patient was active and performed strenuous manual labour that required him to ascend and descend stairs frequently. A SEM3 type (Science et Médecine, Montrouge, France) cementless forged Ti 6Al V4 alloy, with femoral stem size (12) coated with hydroxyapatite on the proximal third, with a metallic head of a diameter 28 mm, and an ultra-high molecular weight polyethylene (UHMWPE) insert (liner) with a metal acetabular cup (50 mm) was inserted. Four years after his original operation (in January 2007), the patient experienced severe pain in the left hip while walking. He was admitted to hospital some days after this episode, with pain in the left hip and inability to bear weight. There was no history of trauma. Radiographic examination revealed a neck fracture of the femoral component without bone loss in the proximal femur (figure 2). For the extraction of the fractured femoral stem a specially designed extractor (patent pending) was invented. Six days after his admission, the patient underwent a revision THA. The elapsed time was needed for the construction of the extractor. At retrieval an extensive amount of bone was adherent to the device, and the fractured implant was well fixed. At revision the same stem design and the same size (12) of the femoral stem was inserted. The metal acetabular cup was not revised but only a new UHMWPE insert was applied because it exhibited areas of attrition due to friction of the fractured stem. The postoperative course was uneventful. The patient was mobilized on crutches for 6 weeks. At follow-up examination, seven months postoperatively the patient remains independently mobile and pain free with a good range of motion. The fractured stem and the neck carrying the head were sent off for stereomicroscope examination with magnifications up to ×40. Images were captured through digital camera (Nikon Coolpix 5400 5 M Pixels or Sony DCR TRV80E 2 M Pixels) and further investigation with scanning electron microscope was decided. The head-side of the neck was thus examined with scanning electron microscope (Quanta 200, FEI Company) and images were taken at magnifications from ×10 to ×110.

**Figure 3:**
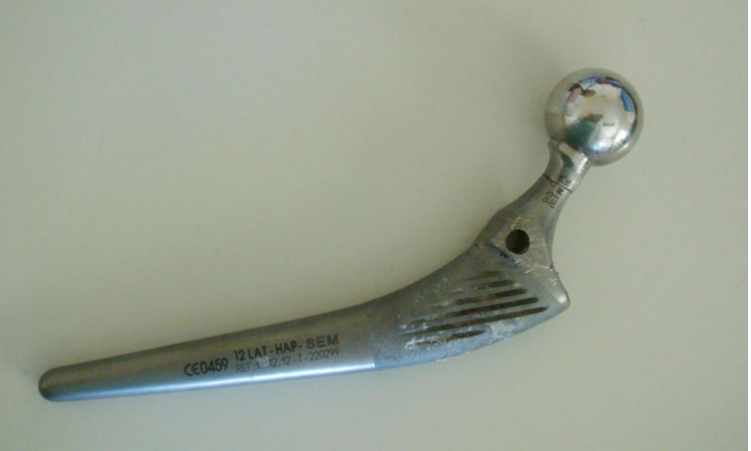
The retrieved stem.

**Figure 4:**
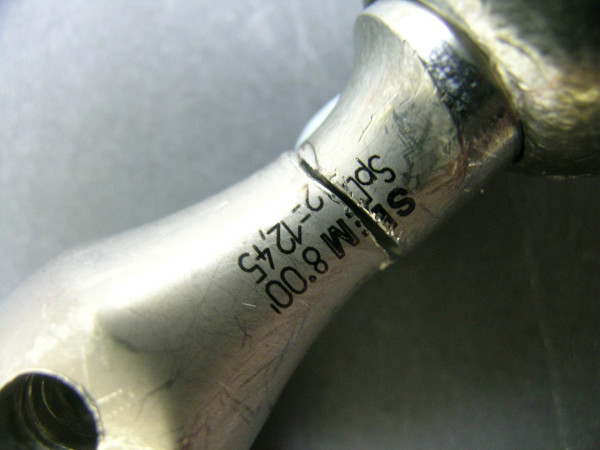
The fractured neck of the femoral implant.

**Figure 5:**
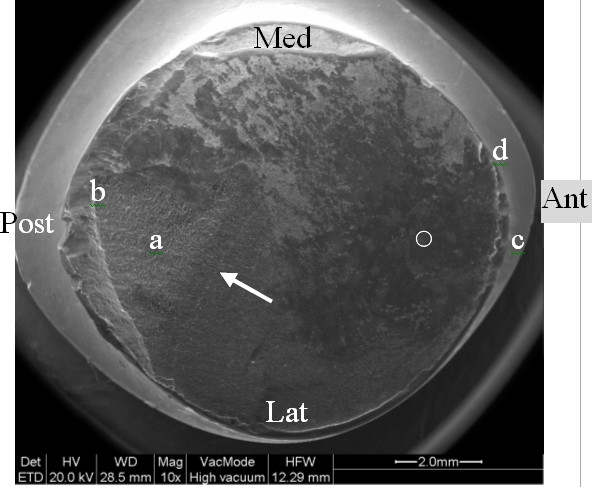
Fracture surface of the head-side. View of the surface fracture of the head side:the arrow indicates the beginning of the intergranular fracture. Regions a and b indicate the location of the intergranular fracture and the shear lip. Regions c and d indicate the location of the anterior laser markings (letters "E" and "D" respectively).

**Figure 6:**
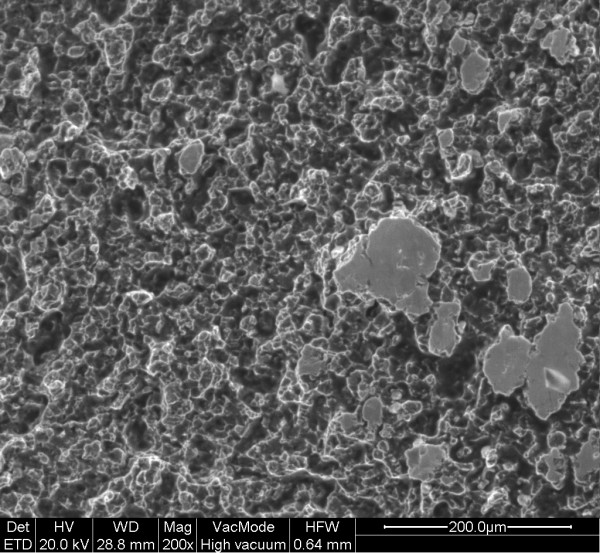
Intergranular fracture (region a).

**Figure 7:**
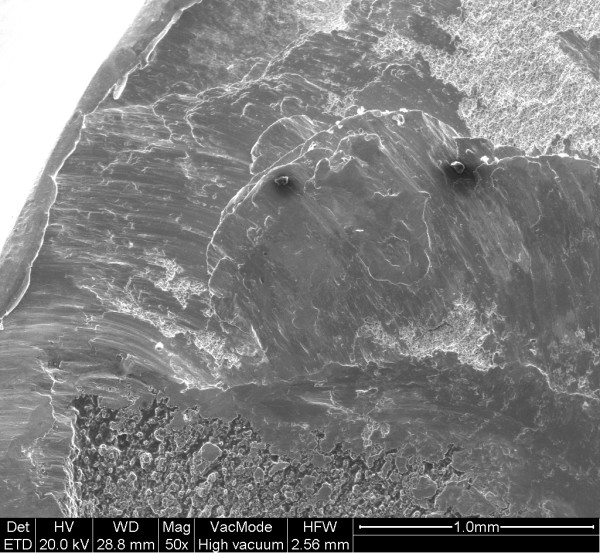
Shear lip (region b).

**Figure 8:**
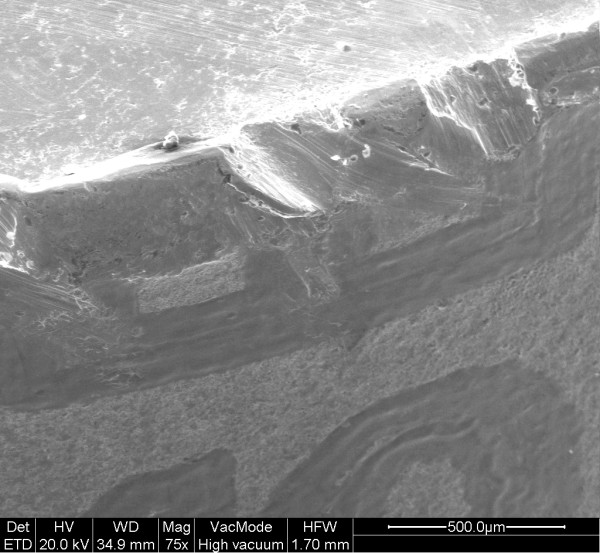
Edge of the fractured surface, in the vicinity of a laser marking (Letter "E", region c).

**Figure 9:**
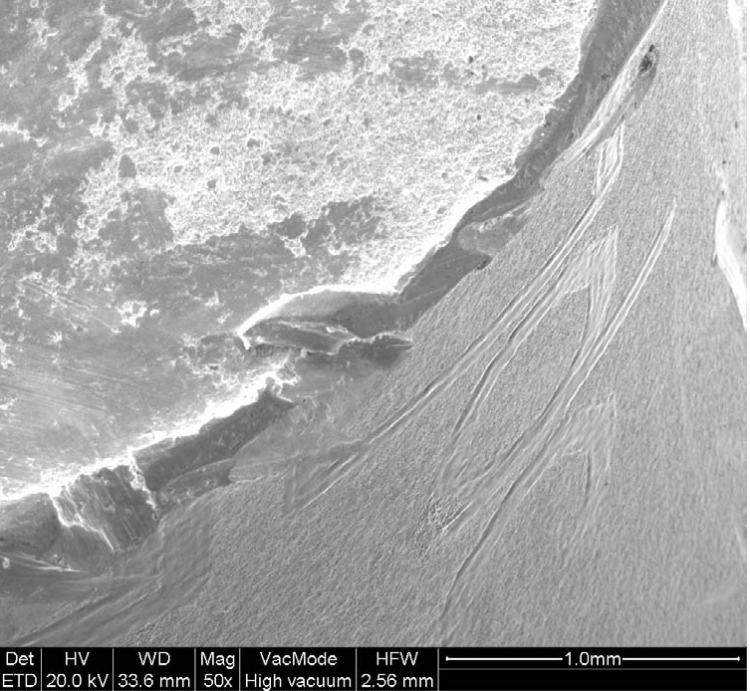
Edge of the fractured surface, in the vicinity of a laser marking (Letter "D", region d).

The examination of the stem and neck (figures 3 and 4) shows that this model has a neck machined for a better range of motion and impingement avoidance, with material removed mainly on the medial side, but machining extends also to the posterior and anterior aspects. Apparently, the fracture occurred at the smallest section of the neck, where the diameter is reduced to approximately 9.8 mm, corresponding to a surface area of 75 mm^2^. The laser etched markings lie on the anterior aspect of the stem, when used in a left hip. Additional laser etched markings are made on the neck, giving the indication relative to the characteristics of the cone (SEM 8. 00' and SpD2 = 12,45). These markings lie also on the anterior aspect, when the stem is implanted on a left hip. Scanning electron microscope figures of the fracture surface of the part supporting the head clearly show that the fracture has ended on the posterior side. Typical beach marks can be seen in the middle of the surface (arrows on figure 5). This beach marks are characteristic of the beginning of intergranular fracture, after a first stage of fatigue crack propagation. The second stage of the fracture consists in an intergranular fracture, located in the region marked a on figure 5, and shown more clearly in figure 6. The very last stage consists in a shear lip (marked region b in figure 5 and figure 7), oriented to 45° relative to the plane of intergranular fracture, and characteristic of the very end of the fracture due to shear stresses. Except for the intergranular fracture, which is very clear, the other fracture surfaces are smeared and polished from abrasive contact in vivo as enhanced by biological lubrication (see fragment contact in figure 2). Examination of the edges of the surface with a stereomicroscope reveals marks of tools, probably iatrogenically caused during retrieval of the implant. Unfortunately, these marks lie on the anterior aspect just in the vicinity of the laser markings, and interfere with possible other marks of fracture. Scanning electron microscope examination of the edge of the surface near the laser markings cannot show any clear evidence of initiation of fatigue fracture, mainly because of the tool marks and of the abraded surface in this region (figures 8 and 9). No typical striations could be found near the edge of the surface. The marks shown in figure 8 (near the "O") are more probably due to attrition because there are several such marks in this region, with different orientations.

## Discussion

Fractures of the femoral stem may occur following primary total hip arthroplasty [1-4]. In comparison with stainless steel, femoral component stems made of high-strength titanium or cobalt-chromium-molybdenum alloys rarely fracture [5,6]. The time in situ of the stem before fracture has been recorded as between 2.5 and 6.5 years [7].

Fractures of the femoral stem have been well documented and may be attributed to one factor or a combination of factors including a) high stresses in the stem due to increased patient weight, a high level of activity, or a relatively undersized prosthesis; b) poor proximal bone support or fixation, which may be due to the absence of the calcar; c) varus orientation of the stem; d) cantilever bending resulting from good distal fixation in the presence of an inadequate proximal cement mantle; e) the presence of a stress riser; and finally f) material defects in the stem itself due to either inadequate design or the fabrication process[8].

Porosity can be generated within the cast during the solidification of the metal, which is accompanied by shrinkage, leaving voids within the metal (termed, shrinkage porosity). Voids can also be generated during expulsion of gases as part of the solidification process (termed, gas porosity).

The grain size is another factor closely related to the fatigue properties of the stem [3]. Historically most stem fractures have occurred at the middle third of the implant, where proximal stem loosening and solid distal stem fixation result in cantilever bending and eventual fatigue failure [9].

Finite-element analysis has shown that the highest stress concentrations are around the lateral aspect of the middle third of the femoral stem. A fracture of the stem usually originates from its antero-lateral aspect [10] corresponding to the tensile stresses generated during walking. Stair climbing and sitting can also generate high tensile stresses on anterior and lateral aspect [2].

Fracture of the neck of the femoral stem is a rare phenomenon, and few have been documented [4,6,11-13]. The femoral neck is a significantly loaded region in total hip prostheses. Crevice corrosion developing at the taper junction between the modular head and neck may lead to an occasional fracture through the neck of the femoral component [6]. Fractures may be due to defects of the welding of the neck to the prosthesis head. This possibility can be excluded by employing a modular component in which welding is not used, but does not exclude other type of problems, such as corrosion [14].

Burstein and Wright [11] reported 2 cases each of cervical fractures in a solid forged, trapezoidal model 28 (t 28) prosthesis. Rand et al [12] reported 3 cases of femoral neck fractures and 32 fractures of the femoral stem following 1808 total hip arthroplasties (T-28 and TR-28). The authors concluded that failure of these implants suggests that significant forces are encountered in this region of a femoral component and suggested that newer modular implant designs must consider these loads. Aspenberg et al [15] reported 5 prosthetic fractures through the neck after 5–15 years and concluded that fractures could be related to the design or the type of materials used. Gilbert et al [6] reported 2 cases of intergranular corrosion-fatigue failure of modular hip implants with a cobalt-alloy head and a cobalt-alloy stem, eighty-five and seventy months after implantation. Both implants failed less than one millimetre distal to the taper junction between the head and the stem (outside of the taper). The fractures occurred at the grain boundaries of the microstructure and appeared to be the result of three factors: porosity at the grain boundaries; intergranular corrosive attack, initiated both at the head-neck taper and at the free surface; and cyclic fatigue-loading of the stem.

Lee et al [9] reported 2 early fatigue failures of well-positioned, well-fixed, cemented, forged, cobalt-chromium femoral components at the neck-shoulder junction. A contributing factor to the implant failures was heavy laser etching in a region of the implant subjected to high stresses, leading to decreased fatigue resistance and subsequent fracture.

Vatani et al [13] reported 9 fractures though the neck of the stem of the prosthesis in 35 total hip arthroplasties using a Charnley type modular 28 head. The fractures occurred after mean 4 (0.3–6) years. All fractures were due to an inadequate confluent radius causing abnormal transmission forces through the neck. The higher the load, as seen in men or in individuals with heavy body weight, the greater the risk of fracture. Metallographic, chemical and micro hardness analyses showed no abnormalities. They ascribed these fractures to faulty design of the stem prosthesis. The multivariate analysis showed that the main independent risk factors for fracture of the femoral component were younger age and heavier weight. The fractures began with a crack in the confluence radius due to the presence of high tension at the indentation in the inferior border of the neck. Variable flexion forces during activity caused propagation of the crack and thus exceeded the resistance of the implant. Morgan-Hough et al in 2004 [4] described a fracture of the neck of a femoral component of a Furlong (JRI Limited, London) fully hydroxyapatite-coated cementless total hip arthroplasty. In our case the stem was a lateralized model, which is a factor that increases the offset of the head, and thus the bending moment generated at the neck. The smallest diameter of the neck is less than 10 mm, and the specific design with removed material on the medial aspect (in order to increase range of motion) tends also to increase the tensile stresses transmitted by the neck. The scanning electron microscope pictures of the fracture surface suggest that the fracture has initiated on the edge of the antero-lateral quadrant, and has ended on the posterior side. The fact that the fracture is not in the latero-medial direction (as expected by a classical stress analysis), suggests that a weakest point has been created somewhere in this region. However it is also known [2] that stair climbing and sitting can generate high tensile stresses in these regions, but these movements are not as frequent as walking. Although the examination of the fracture cannot give for certain the exact location of the initiation process, two potentially weak points can be noted: a) the fact that the fracture passes through the laser markings made on the anterior aspect of the neck can lead us to suspect such type of marking for the initiation mechanism of fatigue fracture. Scanning electron microscopy revealed that the surfaces along the etched characters (Figures 8 and 9) were smoother than the surrounding surface of the implant; this finding is consistent with localized melting from the high-temperature laser beam. The process of laser etching involves temperatures higher than the melting point of the alloy, which can induce localized changes in the metallic microstructure and the creation of a local stress riser, as shown by Woolson [2] in a case report involving fracture initiating from laser etching of the anterior aspect of the stem. Generally the indications concerning the characteristics of the conical junction (angle and smallest diameter at the proximal end of the cone) are marked at the flat top surface of the cone in order to avoid marking a region which is potentially submitted to tensile stresses. Additionally, some manufacturers have abandoned laser marking to the profit of an electrochemical marking which does not induce microstructural alterations of the alloy. This shows that alternative solutions are known and that the type of marking could have been avoided. b) The edge of machining on the medial aspect of the neck (figure 4) expands throughout the length of the neck, from the medial aspect at the cranial section of the neck, to the lateral side at the section where the fracture occurred. Although this radius is rather large at the level of the fractured section, it might act as a stress riser and be involved in the initiation mechanism of the fatigue fracture. A gentle polishing could smooth-off this edge.

## Conclusion

The neck of the femoral stem failed from fatigue. The stereomicroscope and scanning electron studies cannot give evidence of the exact location of the initiation of the fracture, due to iatrogenic marks of tools at the external surface of the fractured neck as well as due to attrition of the fracture surfaces.

The implant failed probably because of a combination of factors: the high demands of the patient (strenuous manual labour), the reduced section of the neck (approximately 9.8 mm, corresponding to a surface area of 75 mm^2^), and the potential stress riser effect of either the laser marking or the edge of neck machining.

Although no additional implant fractures have been reported to the manufacturer, these elements could be useful to improve the design and the manufacturing process of this particular stem, and also to the advice given to the patient concerning levels of activity. Although currently the occurrence of a fractured stem in a primary total hip arthroplasty is rare, in view of advances in design, metallurgy, and cementing techniques, several methods can be used to reduce and even eliminate the likelihood of a prosthetic fracture. Surgical technique should be optimized to improve fixation of the implant to the bone and attention should be paid to the characteristics of the metal used by means of optimal quality control.

## Competing interests

The author(s) declare that they have no competing interests.

## Authors' contributions

TBG was the principal investigator of the study, operated upon the patient, conducted the collection of data and was involved in drafting the article. ODS was involved in drafting the article and in collection of the literature; SAP helped in manuscript drafting and in the collection of the literature; PFB performed the interpretation of stereomicroscopic information, compiled the technical report and was involved in drafting the article; GT, IK, and PA were involved in collection of the literature. All the authors read and approved the final manuscript.

## Consent

Written patient consent was obtained for publication of the report.
